# Prediction of Protein Phosphorylation Sites by Using the Composition of *k*-Spaced Amino Acid Pairs

**DOI:** 10.1371/journal.pone.0046302

**Published:** 2012-10-22

**Authors:** Xiaowei Zhao, Wenyi Zhang, Xin Xu, Zhiqiang Ma, Minghao Yin

**Affiliations:** College of Computer Science and Information Technology, Northeast Normal University, Changchun, People's Republic of China; University of Alberta, Canada

## Abstract

As one of the most widespread protein post-translational modifications, phosphorylation is involved in many biological processes such as cell cycle, apoptosis. Identification of phosphorylated substrates and their corresponding sites will facilitate the understanding of the molecular mechanism of phosphorylation. Comparing with the labor-intensive and time-consuming experiment approaches, computational prediction of phosphorylation sites is much desirable due to their convenience and fast speed. In this paper, a new bioinformatics tool named CKSAAP_PhSite was developed that ignored the kinase information and only used the primary sequence information to predict protein phosphorylation sites. The highlight of CKSAAP_PhSite was to utilize the composition of *k*-spaced amino acid pairs as the encoding scheme, and then the support vector machine was used as the predictor. The performance of CKSAAP_PhSite was measured with a sensitivity of 84.81%, a specificity of 86.07% and an accuracy of 85.43% for serine, a sensitivity of 78.59%, a specificity of 82.26% and an accuracy of 80.31% for threonine as well as a sensitivity of 74.44%, a specificity of 78.03% and an accuracy of 76.21% for tyrosine. Experimental results obtained from cross validation and independent benchmark suggested that our method was very promising to predict phosphorylation sites and can be served as a useful supplement tool to the community. For public access, CKSAAP_PhSite is available at http://59.73.198.144/cksaap_phsite/.

## Introduction

Representing one of the most common protein post-translational modifications (PTMs) in eukaryotes, phosphorylation plays significant roles in a wide range of cellular processes, such as regulation of transcription [1], DNA repair [2], metabolism [3], immune response [4], environmental stress response [5], and cellular motility [6]. Phosphorylation process is catalyzed by a group of enzymes called kinases, which affect certain acceptor residues (serine, threonine and tyrosine) in the substrate sequences. It has been estimated that 30–50% of the proteome undergone phosphorylation [7]. Therefore, accurate recognition of the phosphorylation substrates and the corresponding phosphorylation sites may help fully decipher the molecular mechanisms of phosphorylation related biological processes.

Conventional experimental identification of phosphorylation sites with a site-directed mutagenesis strategy is laborious, expensive, and low-throughput [8]. Recently, the appearance of high-throughput mass spectrometry technique [9] has greatly accelerated the identification of novel phosphorylation sites. Accordingly, several phosphorylation site databases have been established, such as ‘Phospho.ELM’ [10], ‘Phosphorylation Site Database’ [11], ‘PhosPhAT’ [12], and ‘Phosphosite’ [13]. However, some limitations of this technique [14] make the exact prediction of phosphorylation sites difficult, and it always requires very expensive instruments and specialized expertise that are usually not available in general laboratories. With the increasing availability of protein sequence data, there is an urgent need for computational tools that can rapidly and reliably identify phosphorylation sites.

In recent years, many computational predictors have been developed and applied with varying success to predict phosphorylation sites [15]. Most of phosphorylation site prediction tools are kinase-specific, since they need the kinase information of the target proteins as input, such as KinasePhos [16], PPSP [17], NetphosK [18] and GPS [19]. In the establishment of these predictors, proteins collected from the phosphorylation site databases without kinase information were not considered and filtered out. However, it can be found that the majority of experimentally validated phosphorylation sites from the present update of Phospho.ELM dataset did not contain kinase annotations, this part of dataset were thus omitted in the training process of the existing kinase-specific prediction tools. Hence the prediction tools that use the information of kinase annotated proteins can not be regarded as completely perfect for predicting the non-kinase annotated proteins. In other words, these tools are certainly not generalized. In addition, the limitations of kinase-specific prediction tools definitely ignore some important properties of the phosphorylation sites. Therefore, several generalized prediction tools were proposed which ignored the kinase information and only used the primary sequence information for classifying phosphorylation sites, such as DISPHOS [20], Scansite [21], PPRED [22], NetPhos [23], PHOSIDA [11], and AutoMotif Server AMS [24]. More details about these predictors can be found in two recent reviews [25], [26].

In this study, the prediction performance of phosphorylation sites was improved by utilizing a new encoding scheme, *k*-spaced amino acid pairs (CKSAAP), which has been widely used to deal with diverse prediction topics in the field of bioinformatics [27]–[29]. The proposed predictor CKSAAP_PhSite could overcome the limitation by incorporating only sequence information rather than using any kinase specific information. By comparison, the performance of the CKSAAP_PhSite predictor was very promising to predict phosphorylation sites, with a sensitivity of 84.81%, a specificity of 86.07% and an accuracy of 85.43% for serine, a sensitivity of 78.59%, a specificity of 82.26% and an accuracy of 80.31% for threonine as well as a sensitivity of 74.44%, a specificity of 78.03% and an accuracy of 76.21% for tyrosine. CKSAAP_PhSite is a novel phosphorylation site online tool and can provide probability information for prediction results. The online service is freely available at http://59.73.198.144/cksaap_phsite/.

## Methods

### Datasets

The datasets used in this paper were divided into two parts: training dataset and independent testing dataset. The training dataset came from Ashis and co-workers [22]. Experimentally validated phosphorylation sites were extracted from the Phospho.ELM database (version 8.1 released on August 12, 2008) [10], which contained 5725 proteins covering 12373 phosphorylated serine (S) sites, 2525 phosphorylated threonine sites (T) and 1826 phosphorylated tyrosine (Y) sites, these sites were regarded as positive sites (see Text S1). All the remaining S/T/Y residues which were not in a distance of 50 amino acids from any phosphorylated sites of a protein sequence were regarded as negative sites, as done by [22]. The phosphorylated histidine sites were not taken into account in this paper, since the objective of this work was to classify only the most frequently occurred phosphorylated residues. Since the number of phosphorylated sites and the non-phosphorylated sites were highly imbalanced, we repeatedly selected the equal number of negative sites (non-phosphorylated fragments) to match the positive ones (phosphorylated fragments) ten times for each kind of sites (S/T/Y) in the training dataset (see Text S2).

In order to evaluate the prediction performance among different predictors, we collected a new independent testing dataset by extracting the experimentally verified phosphorylated sites from Phospho.ELM which were added after August 12, 2008. Then the redundancy reduction using CD-HIT [30] was performed to ensure that none of the protein sequences showed a sequence similarity of more than 40% within the independent testing dataset and also in the training dataset. Therefore, the independent dataset contained 837 proteins covering 1450 phosphorylated serine sites, 835 phosphorylated threonine sites and 286 phosphorylated tyrosine sites (see Supporting Information Text S3). The negative sites in the independent testing dataset were generated in the same way as in the training dataset.

Similar to the development of other PTM site predictors [31]–[33], the sliding window strategy was utilized to extract positive and negative samples. After a preliminary evaluation, the optimal window size was 27 in this paper, with 13 residues located upstream and 13 residues located downstream of the phosphorylation sites in the protein sequences. In order to ensure a sequence fragment with a unified length, a non-existing amino acid O was used to fill the corresponding positions.

### Construction of feature vectors

In this study, the composition of *k*-spaced amino acid pairs (CKSAAP) based encoding scheme was used. CKSAAP could reflect the characteristics of the residues surrounding phosphorylation sites, and it has been successfully used for predicting palmitoylation sites [34] and mucin-type O-glycosylation sites [35] to represent the sequence fragment. The detailed procedures are described as follows. For a sequence fragment of 2n+1 amino acids, it may contain 441 types (*AA*, *AC*, *AD*, …, *OO*) of *k*-spaced amino acid pairs (i.e. the pairs separated by *k* other amino acids). Then, a feature vector can be described as:
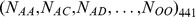
(1)The value of each component is the composition of the corresponding amino acid pairs in the sequence fragment. For example, when there are *m AC* pairs in the sequence fragment, the value of corresponding component *N_AC_* is *m*. After a preliminary evaluation, we found that when the value of *k* increased, the prediction accuracy and the sensitivity would increase, but the computational complexity and the required time for training the models would also increase. So that in this paper, we consider the CKSAAP encoding scheme with *k* = 0, 1, 2, 3, 4 and 5, and the total dimension of the 5-spaced feature vector is 2646. An example of the CKSAAP encoding scheme with *k* = 0, 1, 2, 3 for sequence fragment *AAACD* can be found from Table 1.

**Table 1:** An example of the CKSAAP encoding scheme with *k* = 0, 1, 2, 3 for sequence fragment AAACD.

*K*	*k*-space amino acid pairs	The corresponding feature vectors
0	(*AA*, *AC*, *AD*,…,*OO*)_441_	(2, 1, 0,…,0)_441_
1	(*AXA*, *AXC*, *AXD*,…,*OXO*)_441_	(1, 1, 1,…,0)_441_
2	(*AXXA*, *AXXC*, *AXXD*,…,*OXXO*)_441_	(0, 1, 1,…,0)_441_
3	(*AXXXA*, *AXXXC*, *AXXXD*,…,*OXXX*O)_441_	(0, 0, 1,…,0)_441_

The binary encoding scheme is also carried out here to compare with the CKSAAP encoding. As mentioned above, there are 21 types of amino acids in our setting, which are given as ACDEFGHIKLMNPQRSTVWYO. Therefore, each amino acid is represented by a 21-dimensional binary vector, that is, A corresponds to (100000000000000000000), C corresponds to (010000000000000000000), and O corresponds to (000000000000000000001). For each sequence fragment, the central amino acid is always S/T/Y, which is not necessary to be considered. Consequently, the total dimension of the binary feature vector is 21×2n.

### Feature selection

Because of the high dimensionality of the CKSAAP encoding scheme, a well established filter feature selection method, Information Gain (*IG*) [36] was employed in this paper.

Information Gain is a measure of dependence between the feature and the class label. It is one of the most popular feature selection techniques as it is easy to compute and simple to interpret. Information Gain of a feature *X* and the class label *Y* is calculated as follows:
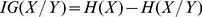
(2)


Information entropy *H*(*X*) is a measure of the uncertainty associated with a random variable (feature) *X*, which is calculated as follows:
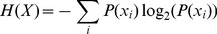
(3)where {*x_i_*} denotes a set of values occurred in *X*, and *P*(*x_i_*) represents the prior probability of *x_i_*. The entropy *H*(*X/Y*) of *X* after observing *Y* is calculated as follows:
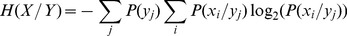
(4)where *P*(*x_i_*/*y_j_*) is the posterior probability of *x_i_* given the value of *y_j_* of *Y*.

For any two features *X_1_* and *X_2_* from the CKSAAP encoding scheme, *Y* is regarded as more correlated with *X_1_* than *X_2_* if *IG*(*X_1_*/*Y*)>*IG*(*X_2_*/*Y*). A feature that gives higher value of *IG* receives higher rank.

### SVM learning

Support vector machine (SVM) is a popular machine learning algorithm mainly used in dealing with binary classification problems. SVM looks for a rule that best maps each member of training set to the correct classification [37], and it has been widely used in bioinformatics community. Formally, given a training vector *x_i_* ∈ *R_n_* and *y_i_* ∈ {−1, +1} be the corresponding class labels, *i* = 1, …, *N*, SVM solves the following optimization problem:
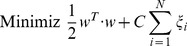
(5)

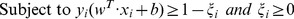
(6)where *w* is a normal vector perpendicular to the hyperplane, the regularization parameter *C* controls the trade-off between the margin and the training error, and 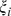 are slake variables for allowing misclassifications [38]. In this paper, LIBSVM package [39] with radial basis kernels (RBF) was used as 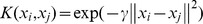 where the kernel width parameter *γ* represents how the samples are transformed to a high dimensional space. In this paper, grid search strategy based on 5-fold cross-validation was utilized to find the optimal parameters *C* and γ ∈ {2^−7^, 2^−6^, …, 2^8^}, so that a total number of 256 grids were evaluated.

### Performance assessment of CKSAAP_PhSite

Three cross validation methods are often used to examine a predictor for its effectiveness: independent dataset test, subsampling test (e.g. 5-fold or 7-fold cross validation), and jackknife test [40]. Of these three test methods, the jackknife test is deemed as the most objective one [41], since the outcome obtained by it is always unique for a given benchmark dataset. However, to reduce the computational time, 5-fold cross validation test is commonly used instead of jackknife test. In the 5-fold cross validation, the dataset is divided into 5 equal subsets, out of which 4 subsets are used for training and the remaining one for testing. This procedure is repeated 5 times and the final prediction result is the average accuracy of the 5 testing subsets. In this study, 5-fold cross validation and independent dataset test are chosen for evaluating the proposed predictor.

In order to evaluate our predictor CKSAAP_PhSite, four measurements are used: sensitivity (*Sn*), specificity (*Sp*), accuracy (*Ac*) and Matthew correlation coefficient (*MCC*). They are defined by the following formulas:
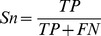


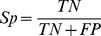


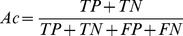


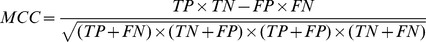
where *TP*, *TN*, *FP* and *FN* stand for the number of true positive, true negative, false positive and false negative, respectively. In addition, the receiver operating characteristic (ROC) curves [42] and the area under the curve (AUC) are also carried out.

## Results and Discussion

### Performance of CKSAAP_PhSite

**Figure 1:**
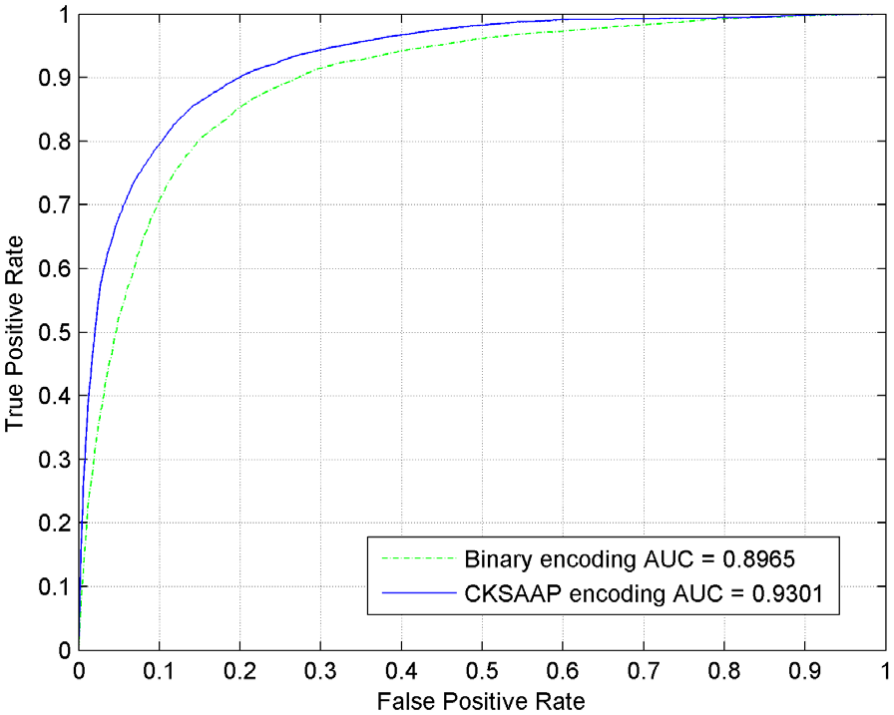
ROC curves of CKSAAP_PhSite and the binary encoding scheme in terms of serine (S) site prediction based on the training dataset.

**Figure 2:**
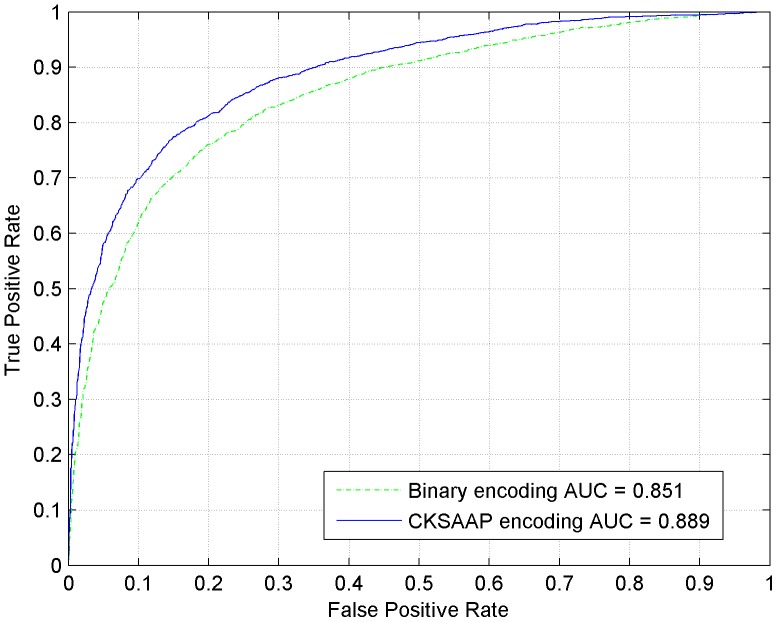
ROC curves of CKSAAP_PhSite and the binary encoding scheme in terms of threonine (T) site prediction based on the training dataset.

**Figure 3:**
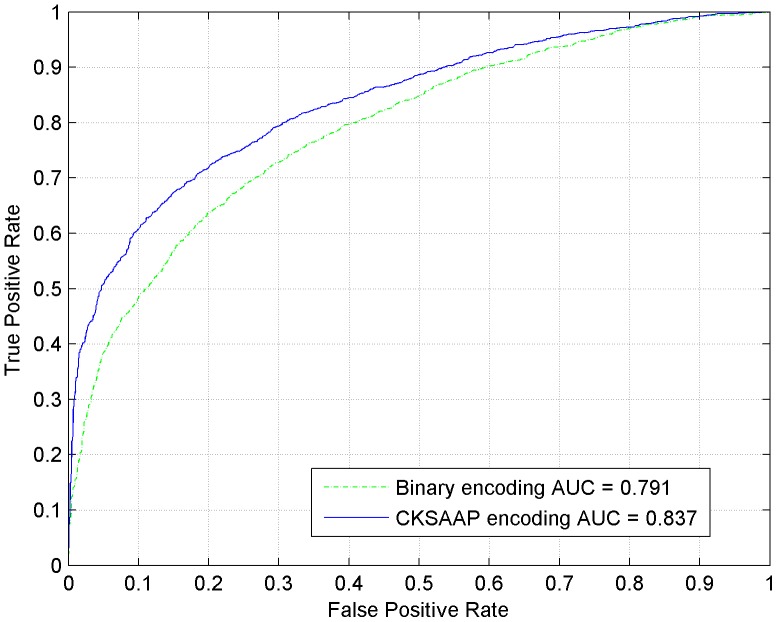
ROC curves of CKSAAP_PhSite and the binary encoding scheme in terms of tyrosine (Y) site prediction based on the training dataset.

For each training dataset, the sequence fragments were firstly encoded as numerical vectors by using the CKSAAP encoding scheme, then the CKSAAP_PhSite predictor was established with the assistance of SVM algorithm. In our experiment, the optimal parameters (*C*, γ) for training S, T, and Y prediction model were (2^2^, 2^−7^), (2, 2^−7^) and (2, 2^−7^), respectively. CKSAAP_PhSite was trained and tested through 5-fold cross-validation, and all of the results were calculated based on the threshold value 0.5. The average performance of CKSAAP_PhSite on the training dataset was summarized in Table 2. The average prediction accuracy (*Ac*) reached 85.43% for S (*Sn* = 84.81%, *Sp* = 86.07%, *MCC* = 0.709), 80.31% for T (*Sn* = 78.59%, *Sp* = 82.26%, *MCC* = 0.599) and 76.21% for Y (*Sn* = 74.44%, *S_p_* = 78.03%, *MCC* = 0.524). Since the proposed CKSAAP_PhSite predictor is a discrete classifier, the ROC curves for each of the three residues (S, T and Y) have been plotted, as can be seen in Figure 1, Figure 2 and Figure 3.

**Table 2:** Comparison of the two encoding schemes on the training dataset.

Site	Encoding scheme	*Sn* (%)	*Sp* (%)	*Ac* (%)	*Mcc*
S	Binary	80.37±0.69	84.89±0.73	82.63±0.61	0.653±0.012
	CKSAAP_PhSite	84.81±0.52	86.07±0.56	85.43±0.82	0.709±0.005
T	Binary	60.05±0.95	90.17±0.64	75.12±0.53	0.528±0.008
	CKSAAP_PhSite	78.59±0.51	82.26±0.86	80.31±0.62	0.599±0.015
Y	Binary	65.07±1.09	81.36±1.06	73.15±0.87	0.471±0.017
	CKSAAP_PhSite	74.44±0.74	78.03±0.21	76.21±0.32	0.524±0.006

Due to the high dimensionality of the CKSAAP encoding scheme, the well established filter feature selection method IG was used to reduce the dimensionality and to find the most relevant features (amino acid pairs). After several rounds of experiments, it was found that the feature selection method resulted in little performance improvement, so that feature selection was not used in the final prediction model. This phenomenon was probably because SVM has a good tolerance to high dimensional data.

### The top ranked features

**Figure 4:**
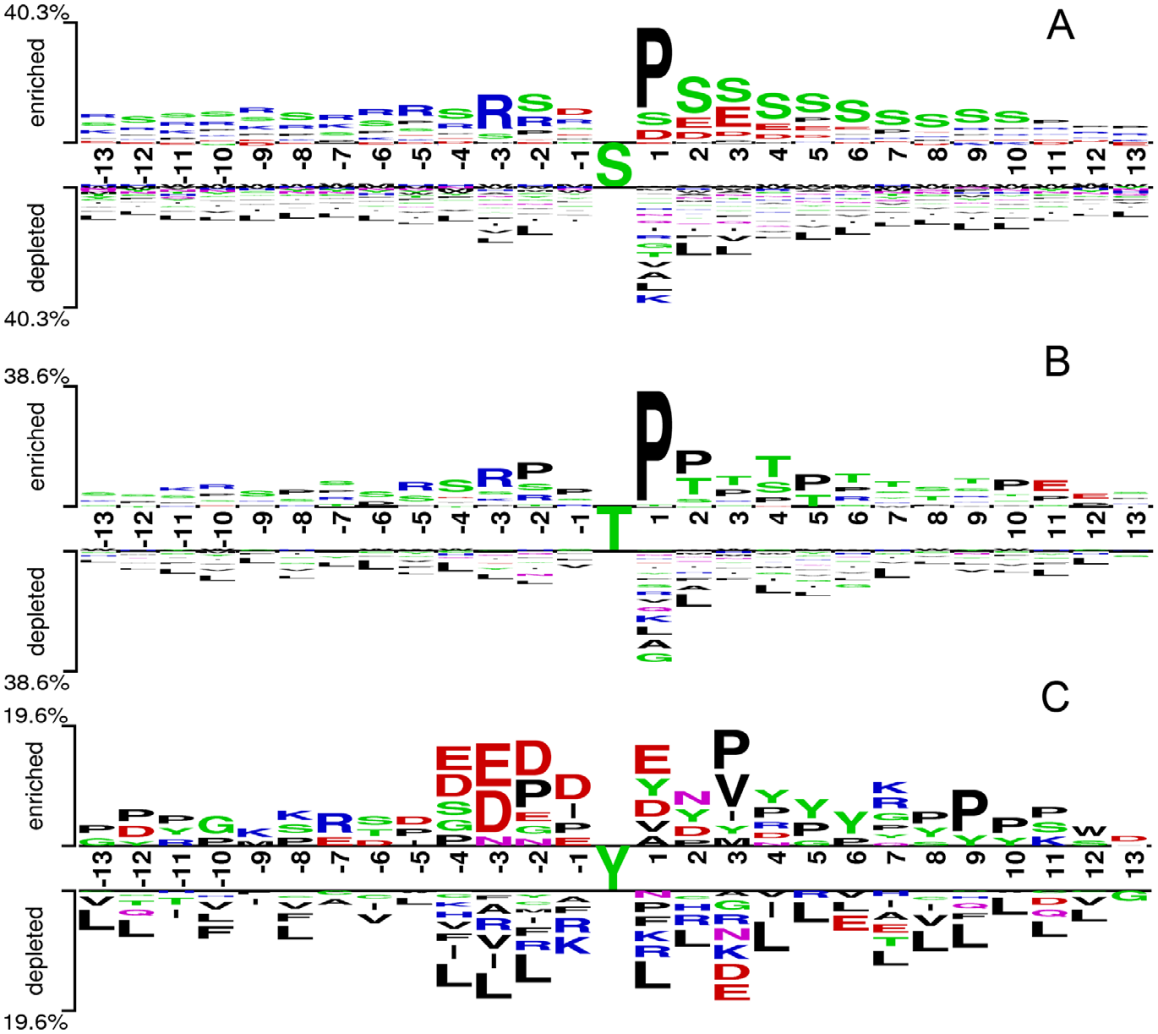
Three Two-Sample-Logos of the position-specific residue composition surrounding the phosphorylated site and non-phosphorylated sites. (A) serine site logo, (B) threonine site logo, (C) tyrosine site logo. These three logos were generated using the web server http://www.twosamplelogo.org/ and only residues significantly enriched and depleted surrounding phosphorylated sites (*t*-test, *P*<0.05) are shown.

Though the feature selection method brought no significant performance improvement, this method could find out the most “important” features (amino acid pairs) generated by the CKSAAP encoding scheme. In order to give some instruments for predicting phosphorylation sites, the top 20 features of phosphorylated S/T/Y sites were listed in Table 3. The importance of these features was also clearly and intuitively characterized in Figure 4. For example, the feature S×S of phosphorylated serine (S) site prediction, which represents the SS residue pair spaced by any amino acid (that is to say, 1-spaced residue pair), is enriched in position pairs surrounding the phosphorylated sites. As can be seen in Table 3, S, T and Y frequently appeared in the top 20 amino acid pairs, which in accord with the observation from Figure 4 that S, T and Y frequently occurred in the vicinity of phosphorylated sites. Table 3 also showed the sequence patterns around the phosphorylated sites, that is, a new sequence fragment including these amino acid pairs in rich would more likely have phosphorylated sites.

**Table 3:** The top 20 features ranked by IG based feature selection method.

The top 20 features	S	T	Y
1	SP	TP	Y××P
2	S×S	P×××P	D×Y
3	S×××S	L××L	L×L
4	R××S	LL	D××Y
5	S××S	SP	LL
6	S×××××S	T××××P	L×××××L
7	S××E	P××××S	DY
8	S××××S	L××××L	V××L
9	S×R	T×P	YE
10	S××××E	T×××T	P×××P
11	SS	L×L	E××Y
12	RS	T×××××P	F×××V
13	R××××S	P××P	L×××L
14	S×××E	PE	P×××××P
15	R×××××S	PP	L×A
16	S×E	L××G	L××L
17	L×××L	S××T	D××S
18	R×××S	P×T	L××××L
19	E×E	RP	P×Y
20	L××L	L×××L	Y×××P

### Comparison with the binary encoding scheme

When compared with the binary encoding scheme on the training dataset, the CKSAAP encoding scheme revealed about 3%, 5%, and 3% higher accuracies for predicting S, T, and Y sites respectively (Table 2). The comparisons were further illustrated by the ROC curves. As can be seen in Figure 1, 2 and 3, CKSAAP encoding was better than the conventional binary encoding. The AUC value resulted from CKSAAP encoding was about 0.03–0.05 higher than that of the binary encoding in all three types of phosphorylation site prediction. When compared with the binary encoding scheme on the independent testing dataset, the AUC value resulted from CKSAAP encoding was about 0.09–0.16 higher than that of the binary encoding in all three types of phosphorylation site prediction.

We also carried out the comparison of CKSAAP and binary encoding based merely on sites containing no ‘O’ residues, the average performance between CKSAAP and the binary encoding was summarized in Table 4. Experimental results showed that the usage of ‘O’ residue could result in slightly different performance. These results also revealed that the using of ‘O’ residue was necessary to make the prediction of the predictor more accurate.

**Table 4:** Comparison of the two encoding schemes on the training dataset containing no ‘O’ residues.

Site	Encoding scheme	*Sn* (%)	*Sp* (%)	*Ac* (%)	*Mcc*
S	Binary	80.29±0.52	84.43±0.54	82.36±0.45	0.648±0.023
	CKSAAP_PhSite	84.41±0.37	85.46±0.48	84.94±0.59	0.699±0.007
T	Binary	62.13±0.28	88.61±0.72	73.82±0.47	0.512±0.004
	CKSAAP_PhSite	78.36±0.67	81.72±0.36	79.59±0.71	0.568±0.028
Y	Binary	66.14±0.87	75.36±0.82	71.04±0.49	0.423±0.021
	CKSAAP_PhSite	72.15±0.63	76.18±0.51	74.16±0.52	0.484±0.005

All the above results explicitly indicated that the CKSAAP encoding has a significant advantage over the binary encoding in predicting phosphorylation sites. This is because that the CKSAAP encoding scheme focuses on the relationship of amino acids at different positions, which can reflect the composition of short linear motif. To our knowledge, a number of PTMs are strongly associated with intrinsic disorder [43]–[47], and many PTMs (e.g. lipidation, GPI-anchor) have been experimentally proved to be correlated with intrinsic disorder regions. Moreover, the short motifs in which two or three residues are conserved often resided in disorder regions [48]. This may be the main reason why the CKSAAP encoding can be better than the binary encoding in predicting phosphorylation sites.

### Comparison with the existing predictors

In this section, the proposed CKSAAP_PhSite was benchmarked against DISPHOS [21], PPRED [20] and NetPhos [23], three of the best phosphorylation site predictors on the independent dataset with 1450, 835 and 286 phosphorylated sites of serine, threonine and tyrosine respectively. The method DISPHOS (DISorder-enhanced PHOSphorylation predictor) [20] used position-specific amino acid frequencies and disorder information to identify phosphorylation sites. PPRED [22] ignored the kinase information and only used the evolutionary information of proteins for classifying phosphorylation sites. NetPhos [23] was a neural network-based method for predicting potential phosphorylation sites, and this predictor did not consider any kinase specific information for prediction.

To conduct a comparison on the independent dataset, all the proteins were predicted via the web servers of CKSAAP_PhSite, DISPHOS, and NetPhos. Due to the absence of online server of the method PPRED, we realized this method using the same ratio of positive to negative samples (1∶1) as PPRED done. For the CKSAAP_PhSite, the final prediction results were average over these ten training datasets for each kind of phosphorylation sites. The performance based on the prediction results were summarized in Table 5, 6 and 7. As shown in Table 5, 6 and 7, the performance of CKSAAP_PhSite was better than DISPHOS, PPRED and NetPhos for all three types of phosphorylated sites prediction on the independent dataset. Each of the comparison tables underlined the competitive performance of the proposed predictor, CKSAAP_PhSite, among all three other existing predictors.

**Table 5:** Performance of DISPHOS, PPRED, NetPhos, and our predictors in terms of serine (S) site prediction on the independent dataset.

Method	Performance parameters of the systems			
	*Sn* (%)	*Sp* (%)	*Ac* (%)	*Mcc*
DISPHOS	81.03	62.86	70.10	0.432
PPRED	72.62	56.42	62.87	0.286
NetPhos	78.90	55.64	64.91	0.343
CKSAAP_PhSite	79.45	78.03	78.59	0.566

**Table 6:** Performance of DISPHOS, PPRED, NetPhos, and our predictors in terms of threonine (T) site prediction on the independent dataset.

Systems	Performance parameters of the systems			
	*Sn* (%)	*Sp* (%)	*Ac* (%)	*Mcc*
DISPHOS	70.06	73.04	71.93	0.421
PPRED	48.26	70.34	62.12	0.187
NetPhos	47.78	74.75	64.70	0.231
CKSAAP_PhSite	79.16	78.88	78.98	0.567

**Table 7:** Performance of DISPHOS, PPRED, NetPhos, and our predictors in terms of tyrosine (Y) site prediction on the independent dataset.

Systems	Performance parameters of the systems			
	*Sn* (%)	*Sp* (%)	*Ac* (%)	*Mcc*
DISPHOS	55.24	74.19	66.62	0.298
PPRED	43.01	65.35	56.42	0.084
NetPhos	45.80	69.30	59.92	0.154
CKSAAP_PhSite	52.10	79.53	68.58	0.329

The better prediction performance of CKSAAP_PhSite may be credited to the appropriate sequence encoding scheme adopted in this manuscript, even though the dimension of the CKSAAP encoding is much higher than the encoding schemes used by other predictors. More importantly, the reasonably good performance of CKSAAP-PhSite implied that the CKSAAP encoding can effectively find out the information of enriched and depleted residue pairs around phosphorylated sites [29].

The proposed predictor (CKSAAP_PhSite) successfully overcame the limitations of the kinase-specific prediction tools in predicting protein phosphorylation sites. In designing the predictor, all the remaining serine, threonine, and tyrosine residues that were reported as phosphorylated sites and which were not located in a distance of 50 amino acids from any of the positive annotated residues were regarded as negative phosphorylated sites. Since information regarding negative phosphorylated sites is scarce, some of these remaining residues may be annotated as phosphorylated sites in future experiments. Therefore, as more validated phosphorylated sites from high throughput proteomic experiments become available, we should re-train the predictor which will in turn enhance the prediction performance.

## Conclusion

Accurate identification of the phosphorylation substrates and the corresponding phosphorylation sites could help fully decipher the molecular mechanisms of phosphorylation related biological processes. Though some researchers have focused on this problem, the overall accuracy of prediction is still not satisfied. In this paper, we have developed a competitive phosphorylation site predictor named as CKSAAP_PhSite from the protein primary sequences. By comparison, the performance of the CKSAAP_PhSite predictor was better than three existing predictors, with a sensitivity of 84.81%, a specificity of 86.07% and an accuracy of 85.43% for serine, a sensitivity of 78.59%, a specificity of 82.26% and an accuracy of 80.31% for threonine as well as a sensitivity of 74.44%, a specificity of 78.03% and an accuracy of 76.21% for tyrosine. Furthermore, feature selection method was used to find out the most “important” features (amino acid pairs). The conclusions derived from this paper might help to understand more of the phosphorylation mechanism and guide the related experimental validation.

Since user-friendly and publicly accessible web-servers represent the future direction for developing practically more useful models, simulated methods, or predictors, a web-server of CKSAAP_PhSite has been developed, which can be freely accessible at http://59.73.198.144/cksaap_phsite/.

## Supporting Information

Text S1 The training dataset contains 5725 proteins covering 12373 phosphorylated serine sites, 2525 phosphorylated threonine sites and 1826 phosphorylated tyrosine sites.
(TXT)

Text S2 Ten sets of randomly selected negative samples for each kind of sites (S/T/Y) in the training dataset.
(TXT)

Text S3 The independent dataset which contains 837 proteins covering 1450 phosphorylated serine sites, 835 phosphorylated threonine sites and 286 phosphorylated tyrosine sites.
(TXT)
